# Modeling and Optimization of Cut Quality Responses in Plasma Jet Cutting of Aluminium Alloy EN AW-5083

**DOI:** 10.3390/ma14195559

**Published:** 2021-09-25

**Authors:** Ivan Peko, Dejan Marić, Bogdan Nedić, Ivan Samardžić

**Affiliations:** 1 Magna Steyr, Liebenauer Hauptstraße 317, 8041 Graz, Austria; 2 Mechanical Engineering Faculty in Slavonski Brod, University in Slavonski Brod, Trg Ivane Brlić Mažuranić 2, 35000 Slavonski Brod, Croatia; dmaric@unsib.hr (D.M.); Ivan.Samardzic@unisb.hr (I.S.); 3 Faculty of Engineering, University in Kragujevac, Sestre Janjić 6, 34000 Kragujevac, Serbia; nedic@kg.ac.rs

**Keywords:** plasma jet cutting, cut quality, aluminium alloy, modelling, optimization

## Abstract

The plasma jet cutting process has a high potential for the machining of aluminium and its alloys. Aluminium is well known as a highly thermally conductive and sensitive material, and because of that there exist uncertainties in defining process parameters values that lead to the best possible cut quality characteristics. Due to that, comprehensive analysis of process responses as well as defining optimal cutting conditions is necessary. In this study, the effects of three main process parameters—cutting speed, arc current, and cutting height—on the cut quality responses: top kerf width, bevel angle, surface roughness *Ra*, *Rz*, and material removal rate were analyzed. Experimentations were conducted on aluminium EN AW-5083. In order to model relations between input parameters and process responses and to conduct their optimization, a novel hybrid approach of response surface methodology (RSM) combined with desirability analysis was presented. Prediction accuracy of developed responses regression models was proved by comparison between experimental and predicted data. Significance of process parameters and their interactions was checked by analysis of variance (ANOVA). Desirability analysis was found as an effective way to conduct multi-response optimization and to define optimal cutting area. Due to its simplicity, the novel presented approach was demonstrated as a useful tool to predict and optimize cut quality responses in plasma jet cutting process of aluminium alloy.

## 1. Introduction

The plasma jet cutting process is a modern non-conventional manufacturing process mostly used in shipbuilding and the metal processing industry. In this process, highly ionized gas containing a very high amount of energy is used to cut different metals such as mild steel, stainless steel, wear- and abrasion-resistant steel, aluminum, copper, etc. at various thicknesses up to 150 mm. Cut quality in this process is mostly affected by different process parameters that are set by a technologist or process engineer. Usually, it is the case that appropriate process parameters settings improve some quality characteristics and worsen others. Due to that, it is desirable to conduct comprehensive research in order to define optimal cutting areas where different cut quality responses simultaneously have optimal solutions. In order to do that, many researchers worldwide performed investigations in order to describe the effects of different process parameters on cut quality responses and to define their optimal values.

Table 1 summarizes some of the recent studies. These studies are classified according to investigated material, analyzed process parameters and process responses, and methods that were applied in order to define significant parameters, describe relations between inputs and outputs and determine optimal process responses values. 

Besides the above literature, a few papers also investigated the machinability and cut quality characteristics in the plasma jet cutting process of aluminium and its alloys [18,19,20,21,22,23,24]. Peko et al. [18] developed an ANN model in order to predict the influence of cutting height, cutting speed, and arc current on kerf width in plasma jet cutting process of aluminium alloy 5083. The ANN model was verified using mean squared error (MSE) and correlation coefficient (R) measures between experimental and predicted responses on validation and testing datasets. After the prediction accuracy of the model was checked, 2D and 3D plots that describe the effect of process parameters on kerf width response were generated. Peko et al. [19] investigated the influence of cutting speed, arc current, and cutting height on dross height in plasma jet cutting of aluminium 5083. In order to define the relations between input parameters and response, mathematical modeling was performed using the fuzzy logic technique. The developed model was checked on the new experimental trials using mean average percentage error (MAPE) and coefficient of determination (R^2^) measures. After validation of the fuzzy logic model, optimal cutting conditions were defined and checked by confirmation experiments. Kadirgama et al. [20] presented a mathematical model for HAZ prediction in air plasma cutting of aluminium alloy 6061. The input process parameters were output current, standoff gap, and pressure. Mathematical modeling was performed by response surface method. A partial swarm optimization algorithm was used for optimization of the HAZ function and to define corresponding process parameters levels that lead to minimal HAZ width. Peko et al. [21] developed an ANN model in order to predict the influence of cutting speed and arc current on the surface roughness *Ra* in the plasma jet cutting process of aluminium alloy 5083. Experimental work was conducted according to a Taguchi L_9_ orthogonal array. The developed ANN model was checked on the datasets for test and validation using R and MSE between experimental and predicted data as validation measures. Finally, based on the mathematical model, 2D and 3D plots were generated in order to analyze the influence of each process parameter as well as their interaction on *Ra* response. From the generated plot, optimal cutting area with minimal surface roughness was approximately defined. Peko et al. [22] researched the influence of process parameters such as cutting speed, arc current, and cutting height on the bevel angle response in the plasma jet cutting of aluminium 5083. Experiments were conducted according to the Taguchi L_27_ experimental plan. The main effects plot and interactions effects plot of the S/N ratio of bevel angle as well as ANOVA were used to define the influence of process parameters and their interactions on the bevel angle. Nearly optimal bevel angle and corresponding process parameters values were defined using the Taguchi optimization approach. Hamid et al. [23] conducted experimental research on aluminium alloy 5083 of thickness 10 mm. They investigated the influence of arc current, feed rate, gas pressure, and cutting distance on surface roughness and conicity responses. The experimental plan was designed according to Taguchi L_9_ orthogonal array. Gray relational analysis was used in order to conduct multi-objective optimization and to approximately define process parameters values that lead to minimal surface roughness and conicity. ANOVA results showed that cutting current and cutting speed are the most significant parameters on analyzed responses. Patel et al. [24] analyzed the influence of arc current, standoff distance, gas pressure, and cutting speed on MRR, top and bottom kerf width, and bevel angle in plasma jet cutting process of aluminium 6082 of thickness 5 mm. In order to discuss the influence of process parameters, main effects plots for each response were generated. These plots define approximately process parameters levels that lead to optimum of each response. ANOVA was performed to define contribution of each process parameter. Results showed that arc current, standoff distance, and cutting speed are significant parameters for all responses.

The above literature review [18,19,20,21,22,23,24] yields the fact that no general computational relationships between input process parameters and multiple cut quality characteristics in plasma jet cutting process of aluminium were defined. The presented papers mostly analyzed influence of process parameters on singular cut quality response such as kerf width [18], dross height [19], HAZ width [20], and surface roughness [21]. The literature review showed that no exact optimization of multiple cut quality responses was performed. In most cases, the optimal cutting area was defined approximately according to generated parameters affects plots [18,19,21], by using Taguchi optimization [22,24] or applying grey relational analysis for the purpose of simultaneous optimization of two process responses [23]. The weakness of the Taguchi method and GRA is in searching for optimal process parameters setting only on discrete parameters values used in the experimental matrix [22,25]. Another conclusion of the literature review concerns the fact that the plasma jet cutting process of the aluminium, especially alloy 5083, has been insufficiently studied and that further research is called for in order to determine an optimum configuration of the process parameters that lead simultaneously to the optimal various cut quality characteristics. Starting from these considerations, this paper conducts the experimental investigation of the plasma jet cutting of aluminium alloy 5083. The research presented in this paper investigates the influence of three process parameters, namely, cutting speed, arc current, and cutting height on the cut quality response: top kerf width, bevel angle, surface roughness parameters *Ra*, *Rz*, and material removal rate. The novelty of this paper is hybrid approach of response surface methodology (RSM) combined with desirability analysis that was used to analyze system’s responses and to conduct simultaneous optimization of cut quality characteristics. In comparison with artificial intelligence (AI) methods and metaheuristic algorithms, this approach is, due to its simplicity, very widely used in various manufacturing industries to effective design optimal settings of process parameters [26]. AI methods such as ANN and fuzzy logic as well as metaheuristic algorithms have higher computational complexity, and they are based on sound knowledge and experience of the problem, as well as creativity and comprehension of ANN and evolutionary mechanisms [27]. In this paper, the generated mathematical models of cut quality characteristics were verified by comparison between experimental and predicted data. ANOVA was applied to determine significance of process parameters and their interactions on analyzed responses. Finally, multi-response optimization was conducted and optimal plasma jet cutting region of aluminium alloy 5083 was defined. Based on that application value of RSM, the desirability of the hybrid approach in investigation of machinability of 5083 alloy in plasma jet cutting process was proved. 

This paper is structured as follows. Section 1 presents the latest research in plasma jet cutting process of different materials. Section 2 describes the experimental setup and experimental results. Section 3 presents mathematical models of cut quality responses, verification of the models, and ANOVA. Discussion of the results is conducted in Section 4. Section 5 presents the results of multi-objective optimization. Section 6 gives appropriate findings and directions for further research in this area.

## 2. Materials and Methods

### 2.1. Experimental Setup

**Figure 1:**
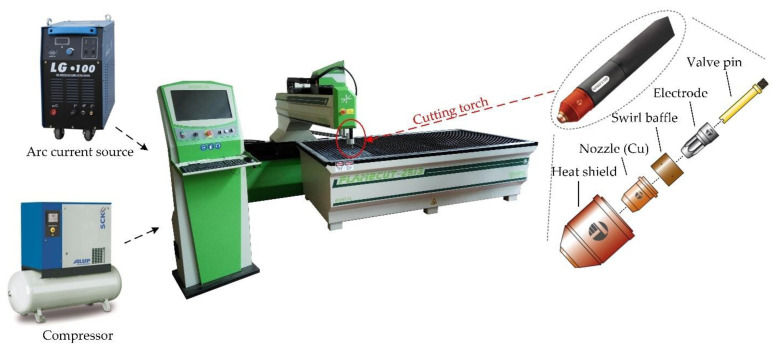
CNC machine FlameCut 2513, cutting torch, arc current source, and compressor.

Experimentations were performed on CNC machine FlameCut 2513 (Arpel Automation, Belgrade, Serbia) with the use of compressed air as plasma gas. The specifications of the CNC machine are given in Table 2. As arc current source an LG 100 IGBT Inverter Air Plasma Cutting Machine was used. For preparing compressed air, a plasma gas compressor (SCK5 200 PLUS, ALUP Kompresoren Gmbh, Reutlingen, Germany) was applied. A purifier and air-drying system were integrated in the compressor. Figure 1 shows the CNC plasma cutting machine together with cutting torch parts, arc current source, and compressor. Workpiece material was aluminium alloy EN AW-5083 H111. Chemical composition, mechanical, and physical properties of workpiece material are presented in Table 3. The aluminium alloy 5083 is a high magnesium alloy with a good strength in the non-heat treatable alloy, good corrosion resistance, and machinability. The arc welding performance is good. The main alloying element in the 5083 alloy is magnesium, which has good corrosion resistance and weldability, as well as moderate strength. Excellent corrosion resistance makes this alloy widely used in marine applications such as ships, as well as in automobiles, aircraft welding parts, and subway light rails [28]. Aluminium is very sensitive to heat input and application of thermal manufacturing technologies such as plasma jet cutting. Due to that, in previous experimental works authors conducted investigation of the influence of variable process parameters on the heat affected zone and material structure [29]. Based on the obtained results, it was concluded that significant structural changes in the heat affected zone did not occur. In fact, here is the case of non-heat-treatable aluminium alloy such as 5083 alloy, which is not sensitive to the heat input during plasma jet cutting process. Significant structural changes are expected to occur during plasma jet cutting and especially welding process of the heat treatable alloys. In the heat-affected zone of these alloys, heat treatment effects would be undone, for the reason of the intermetallic compound precipitates grain size going larger, which would eventually lead to hardness and material strength values decrease [29]. Workpiece sheet thickness is 3 mm. This thickness is very widely used in different industrial applications, especially in shipbuilding for the construction of hulls and marine structures [30]. Experimental trials were conducted according to full-scale experimental design by varying three process parameters on three levels: cutting speed *v* (mm/min), arc current *I* (A), cutting height *H* (mm), as shown in Table 5. Constant process parameters were outlet nozzle diameter: 1.2 mm and plasma gas pressure: 6 bar. The levels of variable and constant process parameters were selected after the detailed literature survey, pilot runs, and industry expert opinions. In each experimental trial, the straight cut length of 80 mm was used.

**Figure 2:**
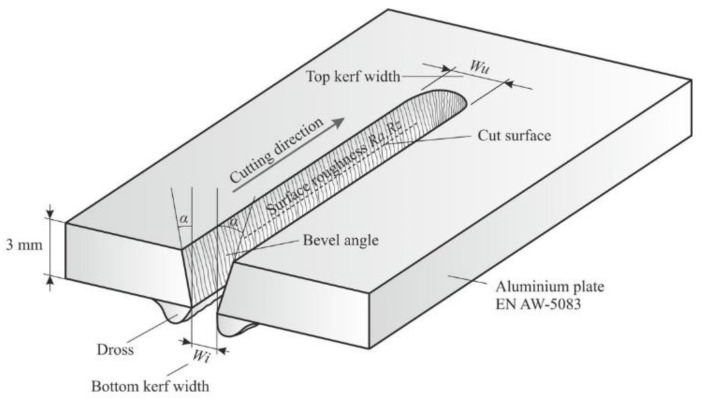
Cut quality responses.

Cut quality responses that were investigated are top kerf width *Wu* (mm), bevel angle *α* (°), surface roughness *Ra* (µm), *Rz* (µm), and material removal rate *MRR* (mm^3^/min), as shown in Figure 2. 

**Figure 3:**
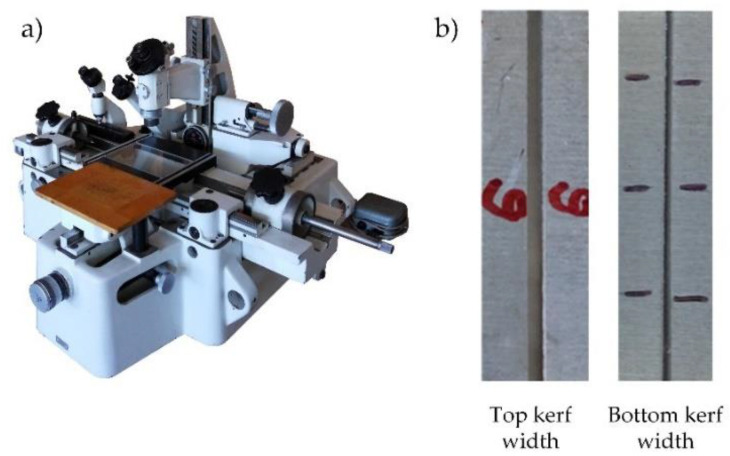
(**a**) Universal Toolmaker’s Microscope and (**b**) example of top and bottom kerf width.

**Figure 4:**
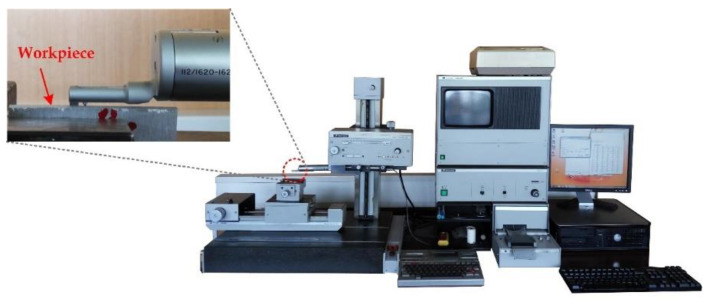
Taylor Hobson Talysurf 6 and example of surface roughness measurement.

Kerf width measurements were made on three equidistant positions along the length of the cut. A Universal Toolmaker’s (Guiyang Xintian Oetech Co., Ltd., Guizhou, China) Microscope was used as a measurement device, as shown in Figure 3a,b, which presents an example of top and bottom kerf width that were measured on the microscope. Specifications of the measurement device are presented in Table 4. Bevel angle was calculated using Equation (1), where *s* (mm) is plate thickness, and *Wu* (mm) and *Wi* (mm) are top and bottom kerf widths, respectively. Surface roughness *Ra* and *Rz* measurements were conducted in the middle of the cut surface height at five equidistant positions along the length of the cut surface. Pre-experiments showed that difference in surface roughness by cut surface height is not visible. Taylor Hobson Talysurf 6 was used as measurement device for surface roughness, as shown in Figure 4. Surface roughness was measured according to standards EN ISO 4287/4288 with the sampling length 8 mm, evaluation length 40 mm, 10 µm radius stylus tip, and Gaussian cut-off filter 8. A stylus speed of 0.5 mm/s was used in conjunction with a 0.8 mN static stylus force and the stylus cone angle used was 90°. In the measurement process, attention was paid on measurement errors that can be random and systematic. In order to avoid random errors measurements of *Wu*, *Ra* and *Rz* were repeated several times until the values converged and average values of each response were considered for the further analysis. Systematic errors were avoided by using correctly calibrated measurement devices in controlled environment under room temperature and pressure. Material removal rate response was calculated according to Equation (2).

(1) $$\alpha \;\left({}^{\circ}\right)=\left|tan^{-1}\left(\frac{Wu-Wi}{2\cdot s}\right)\right|$$

(2) $$MRR\left({\mathrm{mm}}^{3}/\min\right)=\left(\frac{Wu+Wi}{2}\right)\cdot v\cdot s$$

### 2.2. Experimental Results

Full-scale experimental design and cut quality responses values for each experimental trial are listed in Table 5. 

### 2.3. Modelling and Optimization

The process mathematical model defines relations between input parameters and process responses. Therefore, it is possible to predict the response for each input parameter value. In this paper, relationships between process parameters and cut quality responses were determined by developing a regression based mathematical models. The regression models were generated using experimental data from Table 5 in software MINITAB 17. Normally, a second-order polynomial is applied to form mathematical models. The second-order model for three parameters is given in Equation (3), where *Y* is cut quality response: top kerf width; bevel angle; surface roughness *Ra*, *Rz* and material removal rate; *X*_1_, *X*_2_ and *X*_3_ are coded values of cutting speed, arc current, and cutting height; and *B_0_*, *B_1_*, *B_2_* etc. represent the regression coefficients that need to be determined. Once the model is generated, the coded values of the input process parameters need to be substituted in the equation to get the predicted response. In order to convert process parameters from real values in coded values, and vice versa, Equation (4) can be used, where *p_max_*, *p_min_* represent maximal and minimal real process parameters values, *p_real_* real process parameters values, and *p_cod_* coded process parameters values.

(3) $$Y=B_{0}+B_{1}X_{1}+B_{2}X_{2}+B_{3}X_{3}+B_{11}X_{1}^{2}+B_{22}X_{2}^{2}+B_{33}X_{3}^{2}\;+B_{12}X_{1}X_{2}+B_{13}X_{1}X_{3}+B_{23}X_{2}X_{3}$$

(4) $$\;p_{real}=\left(\frac{p_{max}+p_{min}}{2}\right)+\left(\frac{p_{max}-p_{min}}{2}\right)\cdot p_{cod}\to p_{cod}=2\cdot \left(\frac{p_{real}-p_{min}}{p_{max}-p_{min}}\right)-1\;$$

Once the regression models of cut quality responses are created they need to be validated. Validation was conducted in comparison between experimental responses and those predicted by mathematical functions. As validation measures, mean absolute percentage error (MAPE) and coefficient of determination (R^2^) was used. MAPE was calculated according to Equation (5) and R^2^ was calculated according to Equation (6), where *y_i_* is response *y* value for observation *i*, $\bar{y}$ is the mean of response *y* value, ${\hat{y}}_{i}$ is predicted value of response *y* for observation *i*, and SSR is sum of squared regression also known as variation explained by the model, calculated according to Equation (7). SST is the sum of squared total also known as total variation in the data, calculated according to Equation (8). MAPE and R^2^ are widely used in many researches as good measures to check prediction accuracy of developed mathematical models [31,32,33,34,35].

(5) $$MAPE=\frac{100\%}{n}\overset{n}{\underset{i=1}{\sum }}\left|\frac{y_{i}-\hat{y_{i}}}{y_{i}}\right|$$

(6) $$R^{2}=\frac{SSR}{SST}\;$$

(7) $$SSR=\underset{i}{\sum }{\left(\hat{y_{i}}-\bar{y}\right)}^{2}$$

(8) $$SST=\underset{i}{\sum }{\left(y_{i}-\bar{y}\right)}^{2}$$

In order to find out process parameters values that lead simultaneously to minimal top kerf width, minimal bevel angle, minimal surface roughness *Ra*, *Rz* and maximal material removal rate multi-objective optimization of cut quality responses was conducted. Optimization was performed using desirability analysis in MINITAB 17 software. Desirability analysis is technique introduced by Derringer and Suich [36] that is useful to solve optimization problems in industry especially those with multi-response quality characteristic situation [36,37]. Desirability analysis technique transforms each predicted response to a dimensionless value called individual desirability, which varies over the range of 0 to 1. If the desirability value is 1, then response is on its target, and if it is 0 then the response value is outside of acceptable range. In the case of multi-response (multi-objective), optimization composite desirability is calculated. Composite desirability is the weighted geometrical mean of the individual desirability of each response [37]. Composite desirability function will be maximized or minimized depending on process responses characteristics that need to be optimized using optimization algorithm. Composite desirability depends on the weight and importance of each response that is considered in process optimization. The weight determines the shape of the desirability function and the way that the desirability is distributed in the interval between the lower or upper bound and the target [38]. Weight can be determined in the range from 0.1 to 10 to emphasize or de-emphasize the importance of hitting the target value. If the weight is 1, then it is a neutral setting that represents equal importance on the target and on the bounds. If weight > 1, then more emphasis is on the target, and vice versa, if weight < 1 less emphasis is on the target [38]. The importance determines the relative importance of multiple responses. Importance values are between 0.1 and 10. If all responses are equally important then default value of 1 should be selected. Larger values of importance represent more important responses, smaller values less important responses [39].

## 3. Results

### 3.1. Top Kerf Width 

The regression model for prediction of top kerf width *Wu* in coded form is given in Equation (9):

*Wu* = 1.9793 − 0.0030 *v* + 0.0793 *I* + 0.0407 *H* + 0.2140 *v*^2^
*+* 0.1003 *v·I +* 0.0539 *I·H* (*R*^2^ = 0.822) (9)

**Figure 5:**
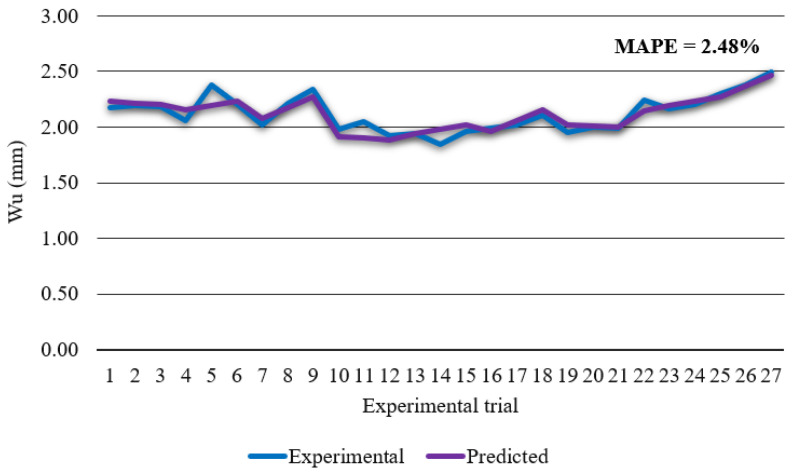
Experimental top kerf width vs. predicted top kerf width.

Prediction accuracy of developed model was checked by comparison between predicted and experimental top kerf width data. Figure 5 shows that developed regression model has high accuracy in estimating top kerf width with a calculated MAPE of 2.48% and *R*^2^ of 0.822. In order to check significance of each parameter on top kerf width ANOVA was conducted. ANOVA results are presented in the Table 6.

**Figure 6:**
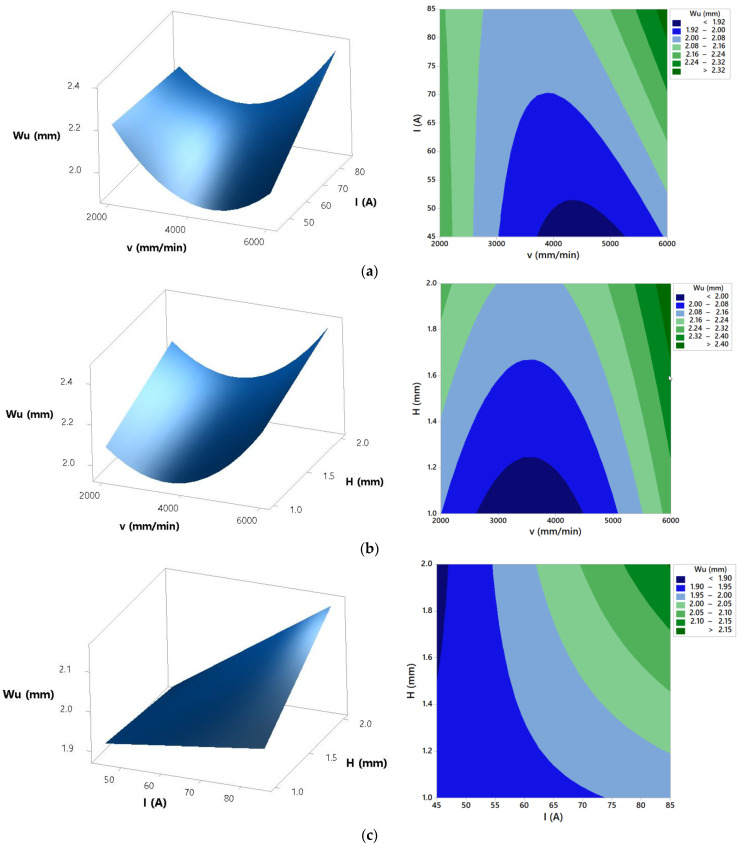
Effects of process parameters on top kerf width: (**a**) *H* = 1.5 mm, (**b**) *I* = 85 A, and (**c**) *v* = 4000 mm/min.

Using developed regression model response surfaces and contour plots that show the effects of process parameters on top kerf width were generated, as shown in Figure 6. 

### 3.2. Bevel Angle

The regression model for prediction of bevel angle *α* in coded form is given in Equation (10):

*α* = 5.847 + 2.529 *v* − 2.369 *I* − 0.313 *H* + 1.115 *v*^2^ + 0.807 *I*^2^ − 0.803 *H*^2^ + 0.415 *v·H* (*R*^2^ = 0.957) (10)

**Figure 7:**
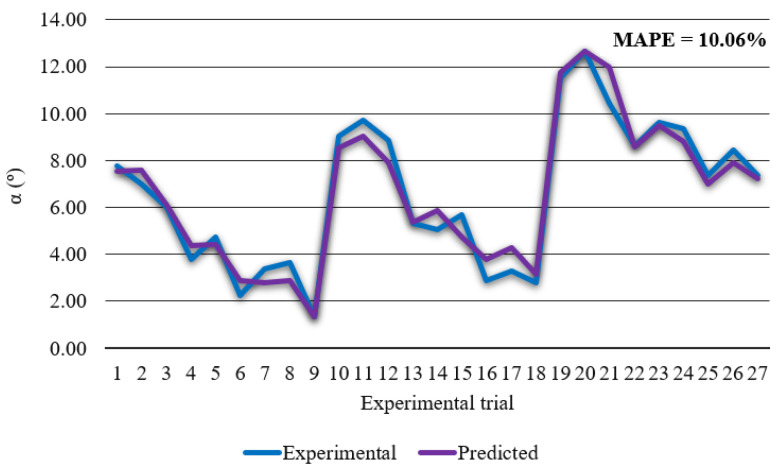
Experimental bevel angle vs. predicted bevel angle.

Figure 7 shows that developed regression model has high bevel angle prediction accuracy with the MAPE of 10.06% and *R*^2^ of 0.957. In order to check significance of each parameter on bevel angle, ANOVA was conducted. ANOVA results are presented in Table 7. 

**Figure 8:**
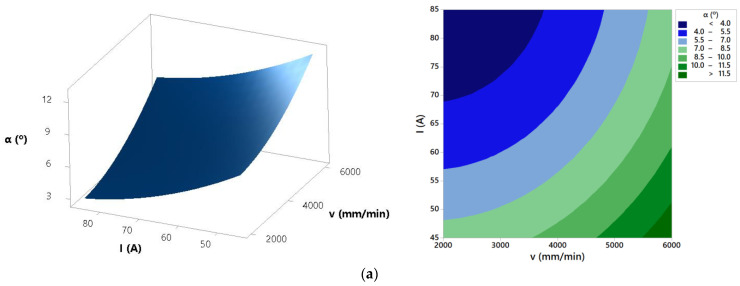

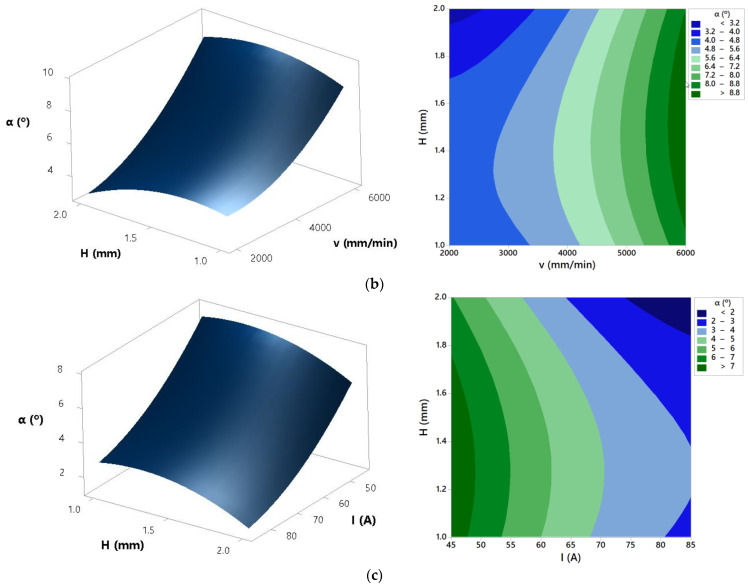
Effects of process parameters on bevel angle: (**a**) *H* = 1.5 mm, (**b**) *I* = 65 A, and (**c**) *v* = 2000 mm/min.

From the developed mathematical model, response surfaces and contour plots of the effects of process parameters on the bevel angle were derived. These plots are shown in Figure 8. 

### 3.3. Surface Roughness Ra

The regression model for prediction of surface roughness *Ra* in coded form is given in Equation (11):

*Ra* = 9.685 − 4.744 *v* + 0.150 *I* − 0.400 *H* + 1.756 *v*^2^ + 1.506 *I*^2^ − 2.708 *v·I* (*R*^2^ = 0.935) (11)

**Figure 9:**
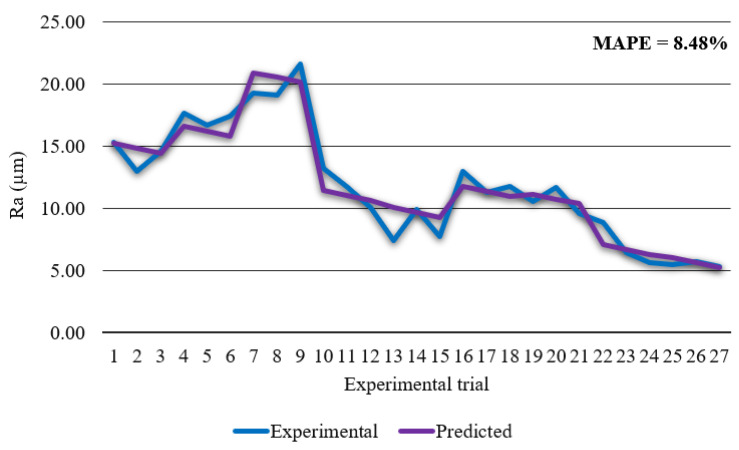
Experimental surface roughness *Ra* vs. predicted surface roughness *Ra*.

From Figure 9 it is visible that developed regression model has high prediction accuracy of surface roughness *Ra* with the MAPE of 8.48% and *R*^2^ of 0.935. ANOVA was performed in order to check significance of process parameters and their interactions on surface roughness *Ra*. ANOVA results are shown in Table 8.

**Figure 10:**
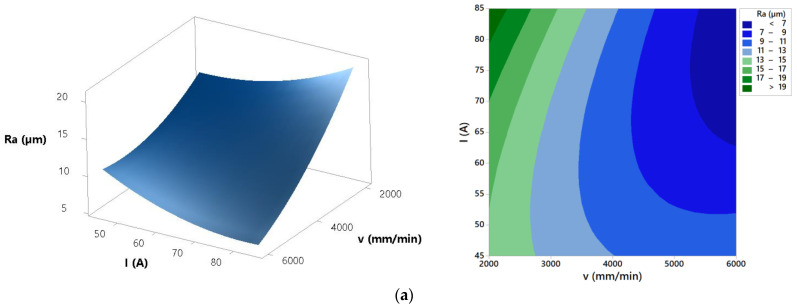

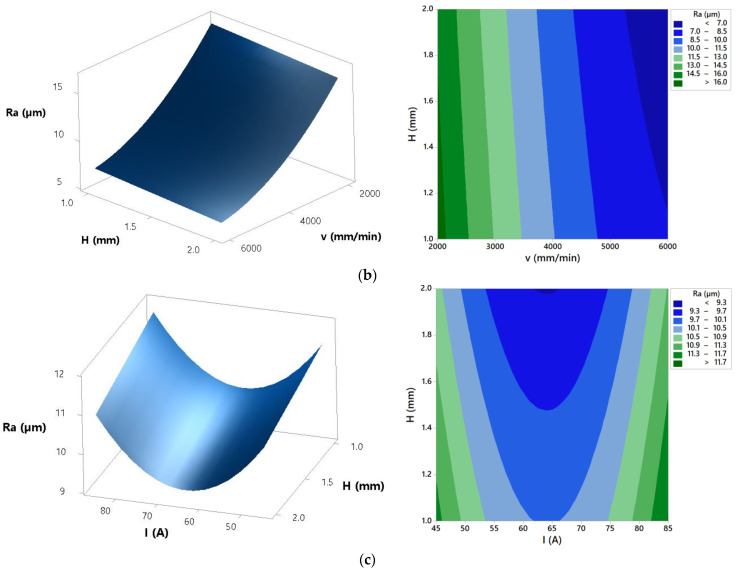
Effects of process parameters on surface roughness *Ra*: (**a**) *H* = 1.5 mm, (**b**) *I* = 65 A, and (**c**) *v* = 4000 mm/min.

Using developed mathematical model response surfaces and contour plots that show the effects of process parameters on the surface roughness *Ra* were generated. These plots are presented in Figure 10. 

### 3.4. Surface Roughness Rz

The regression model for prediction of surface roughness *Rz* in coded form is given in Equation (12):

*Rz =* 46.93 − 16.72 *v* − 0.39 *I* − 0.50 *H* + 4.94 *v*^2^ + 7.28 *I*^2^ − 7.50 *v·I* (*R*^2^ = 0.854) (12)

**Figure 11:**
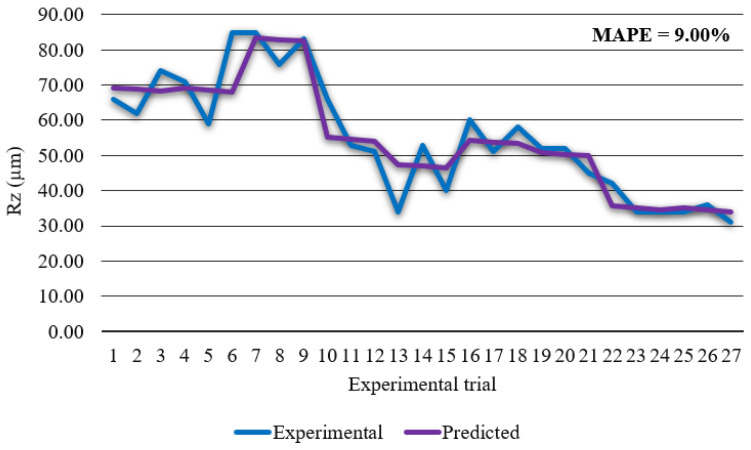
Experimental surface roughness *Rz* vs. predicted surface roughness *Rz*.

From Figure 11 it can be derived that mathematical model for surface roughness *Rz* has high prediction accuracy with calculated MAPE of 9.00% and *R*^2^ of 0.854. In order to define contribution of each process parameter or even their interactions ANOVA was performed. Results of ANOVA are visible in Table 9. 

**Figure 12:**
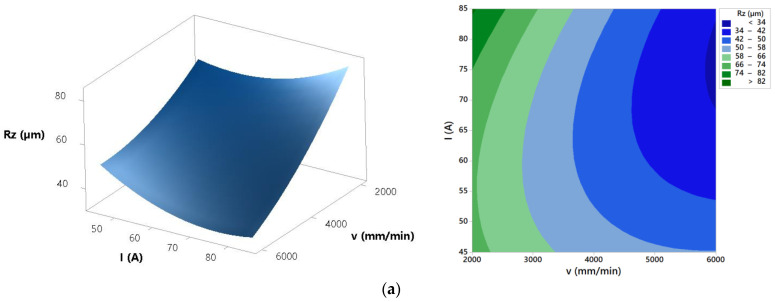

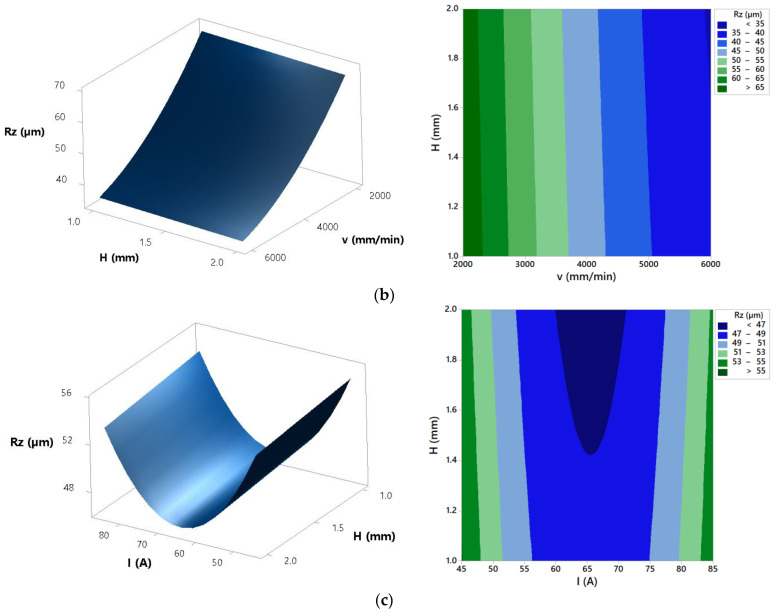
Effects of process parameters on surface roughness *Rz*: (**a**) *H* = 1.5 mm, (**b**) *I* = 65 A, and (**c**) *v* = 4000 mm/min.

The mathematical model was applied to generate plots which present the effects of process parameters on surface roughness *Rz*. Response surfaces and contour effects plots are shown in Figure 12.

### 3.5. Material Removal Rate

The regression model for prediction of material removal rate *MRR* in coded form is given in Equation (13):

*MRR* = 20,056 + 9012 *v* + 3144 *I* + 436 *H* + 1318 *v*^2^ + 1991 *v·I* (*R*^2^ = 0.987) (13)

**Figure 13:**
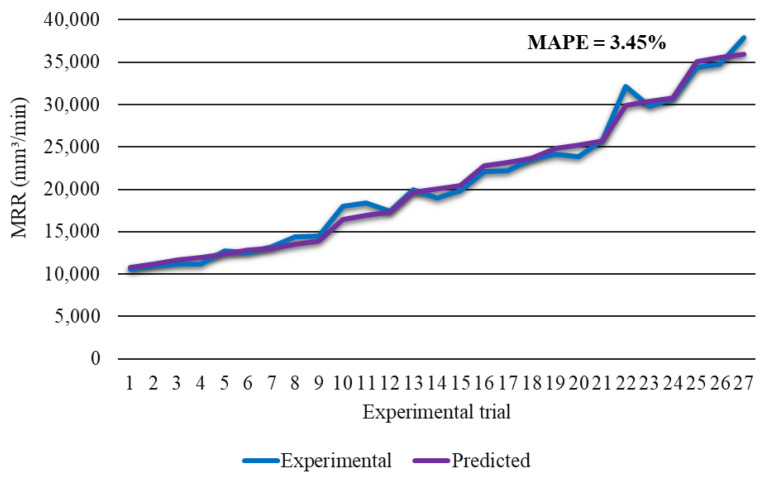
Experimental material removal rate vs. predicted material removal rate.

Figure 13 confirms high prediction accuracy of developed regression model for material removal rate with calculated MAPE of 3.45% and *R*^2^ of 0.987. In order to define significance of process parameters and their interactions, ANOVA was carried out. Results of ANOVA are presented in Table 10.

**Figure 14:**
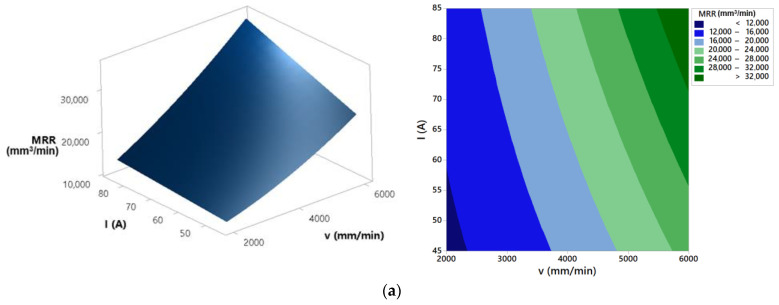

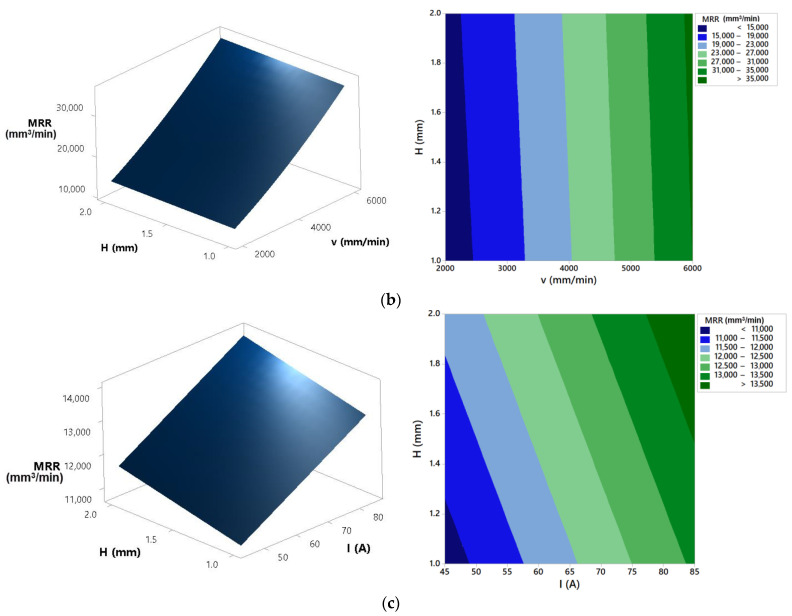
Effects of process parameters on material removal rate: (**a**) *H* = 1.5 mm, (**b**) *I* = 85 A, and (**c**) *v* = 2000 mm/min.

Generated model was used to analyze the effects of process parameters on the material removal rate. In order to do that, response surfaces and contour plots were created, as shown in Figure 14. 

## 4. Discussion of Parameters Effects on Process Responses

From the presented results, for all process responses it can be derived that the developed mathematical models showed high prediction accuracy, and due to that they can be further applied in analysis of process parameters effects. ANOVA for top kerf width presents that the most significant parameters on the top kerf width are cutting speed, arc current, and their interaction. Cutting height as well as interactions cutting speed × cutting height and arc current × cutting height have an insignificant effect on the top kerf width. The effects of cutting speed, arc current, and cutting height are shown in Figure 6. Response surfaces and contour plots from Figure 6 highlight that increase of the arc current results with the increase of the top kerf width. Regarding the cutting speed effect, Figure 6b) presents that the lowest (2000 mm/min) and the highest (6000 mm/min) cutting speeds lead to the wider kerf at the arc current of 85 A. In [6], the results of analysis showed that higher cutting speeds produce an erratic arc, creating a deviation of the arc from the axis of the torch producing a larger kerf. In [40], it was established that cutting heat input is proportional to the arc current and arc voltage multiplication and inversely proportional to the cutting speed. According to that, higher arc current and lower cutting speed lead to the higher heat input, resulting in the increase of the top kerf width, as shown in Figure 6b. Figure 6c shows that increase of the cutting height at the higher arc currents (>65A) and cutting speed of 4000 mm/min leads to the increase of the top kerf width. The same conclusions were derived in [6]. A higher value of standoff distance creates a lack of arc coherence leading to deflection of the arc and producing a larger kerf [6]. According to the derived conclusions, the lowest values of the top kerf width can be achieved when cutting process is defined in the area of cutting speed of 4000 mm/min and lower arc currents (<65 A). 

**Figure 15:**
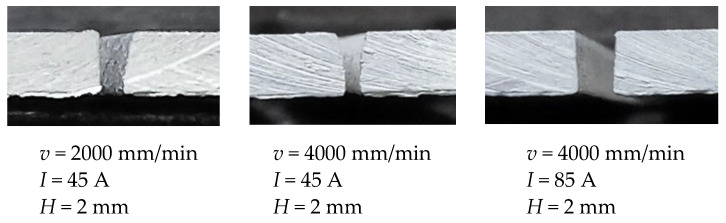
Bevel angles obtained at different process parameters values.

The ANOVA for bevel angle presents that all three parameters as well as interactions cutting speed × arc current and cutting speed × cutting height have a significant effect on the analyzed response. Interaction arc current × cutting height does not have a significant influence on the bevel angle. Bevel angle determines the difference between top kerf width and bottom kerf width. The smaller the bevel angle is, the more perpendicular cut is and the better cut quality is. The response surfaces and contour plots from Figure 8 show that the increase in the cutting speed and the decrease of the arc current result in the increase of the bevel angle. In [41], it was also demonstrated that with an increase in cutting speed and a decrease of the arc current, cutting energy decreases. Accordingly, kerf widths decrease while the bevel angle increases due to a faster decrease of bottom kerf width than top kerf width. From Figure 8 it is visible that increase of the cutting height leads to the slightly smaller bevel angle. As explained in [6], higher cutting height results with the plasma arc deflection that leads to the wider top and bottom kerf width and minor difference between them. Finally, this generates a decrease in the bevel angle. In order to get minimal bevel angle values, plasma jet cutting process should be defined in the area of the lower cutting speed (<4000 mm/min), higher arc currents (>65 A) and higher cutting heights (>1.5 mm). Above presented conclusions are confirmed with the Figure 15. Figure 15 presents bevel angles obtained in the experimental trials at different process parameters values. 

**Figure 16:**
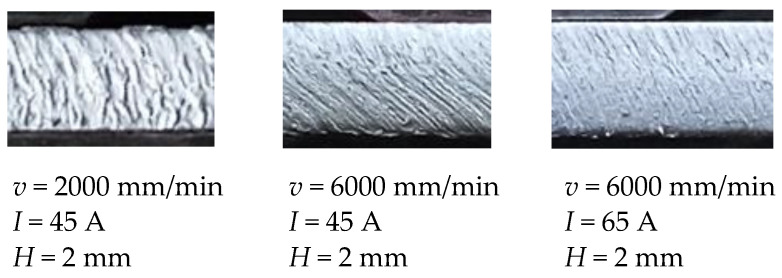
Surface roughness obtained at different process parameters values.

Regarding surface roughness characteristics *Ra* and *Rz* ANOVA highlights that cutting speed and arc current as well as their interaction have significant effect on the process response. Other parameters and interactions do not affect significantly surface roughness characteristics *Ra* and *Rz*. Cut surface roughness formation in plasma jet cutting process is mainly induced by molten metal fluctuation, flow perturbation of plasma jet, cutting torch vibration and motion of anode spots within the groove of cut [40]. Response surfaces and contour plots from Figure 10 present that increase of the cutting speed results with the decrease of the surface roughness *Ra* and *Rz*. Increase of the cutting speed leads to the more intensive fluctuation of the molten metal that results with the lower surface roughness of the cut. Furthermore, at lower cutting speeds motion of anode spots appears and that results with the higher roughness [40]. The same trends were shown in [6,14,40]. Regarding arc current effects from Figure 10 it is visible that at low cutting speed (2000 mm/min) increase of the arc current results with the higher cutting energy and increase of the surface roughness. Fluctuation of the molten metal from the groove of the cut is less intensive and that leads to the rougher cut surface [40]. At high cutting speed (6000 mm/min) increase of the arc current leads to the slightly decrease of the surface roughness. Higher cutting energy combined with the more intensive fluctuation of the molten metal result with the lower surface roughness [40]. Figure 16 shows surface roughness obtained at different process parameters values. According to the above derived assertions in order to achieve minimal surface roughness *Ra* and *Rz* plasma jet cutting process needs to be concentrated in the area of the higher cutting speeds (>4000 mm/min) and higher arc currents (>65 A).

ANOVA results for material removal rate present that the most significant parameters that affect process response are cutting speed, arc current, and their interaction. Cutting height and interactions cutting speed × cutting height and arc current × cutting height have an insignificant effect on the material removal rate. Material removal rate is proportional to top and bottom kerf width, cutting speed, and workpiece thickness (Equation (2)). Response surfaces and contour plots from Figure 14 show that increase of the arc current results with the increase of the material removal rate. A high concentration of plasma energy is transferred to the workpiece at higher arc current and that leads to quick melting and the vaporization of the metal and higher material removal rate [17]. At higher cutting speeds the unstable plasma arc is formed [6,40]. That leads to the larger top and bottom kerf width and accordingly higher material removal rate. From Figure 14 it is visible that cutting height has negligible effect on the material removal rate response. However, increase of the cutting height results with the slightly increase of the material removal rate. As it was already stated in [6], higher cutting height results with the deflection of the arc due to the lack of arc coherence. That leads to the wider kerf and higher material removal rate.

## 5. Multi-Objective Optimization of Cut Quality Responses

In order to find out process parameters values that lead simultaneously to optimal cut quality responses of minimal top kerf width, minimal bevel angle, minimal surface roughness *Ra*, *Rz* and maximal material removal rate, multi-objective optimization was conducted. In order to avoid bias in importance estimation of each response multi-objective optimization was performed using neutral settings of weights and identical importance value for each output. That means that each process response is the equally important. Practically, importance of each process response can be defined subjective depending on current industrial requests for achieving better or worse value of some cut quality characteristics. According to that, different optimization results will be provided [27].

In Table 11, multi-objective optimization results and respective objectives are listed. It is presented set of five Pareto-optimal solutions. Pareto-optimal solutions represent compromise solutions that satisfy different opposed objective functions (min *Wu*, min *α*, min *Ra*, min *Rz*, max *MRR*).

**Figure 17:**
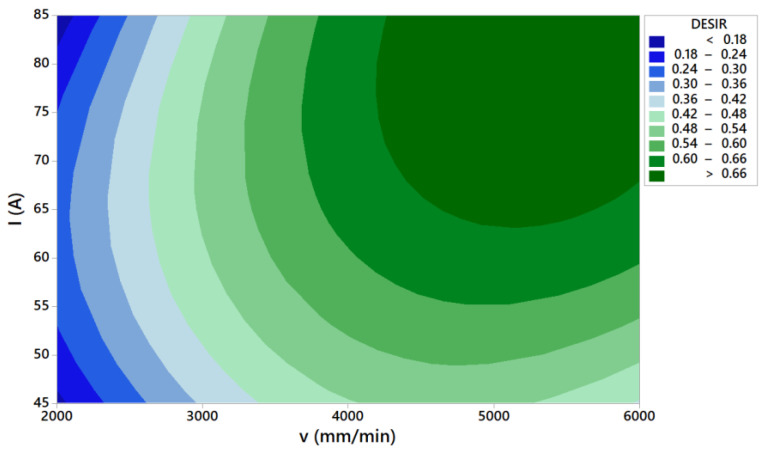
Desirability contour plot for multi-objective optimization *Wu*-*α*-*Ra*-*Rz*-*MRR* at *H* = 1 mm.

**Figure 18:**
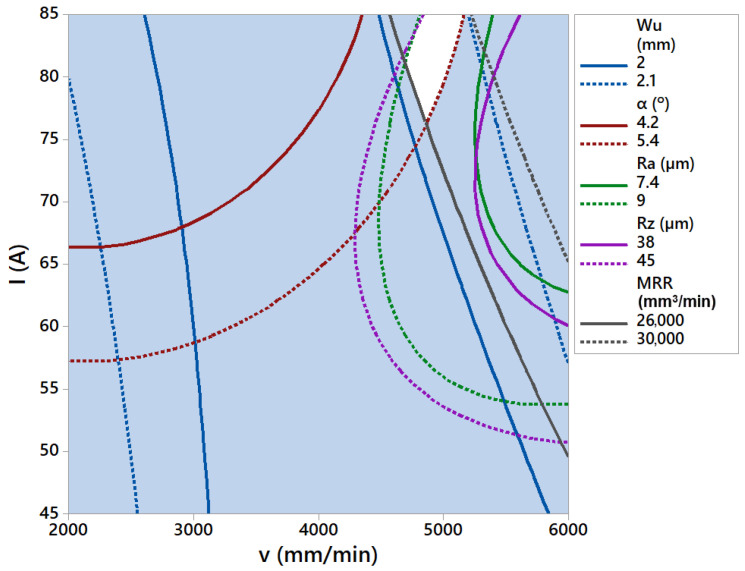
Optimal cutting area (white colored) at *H* = 1 mm.

Figure 17 shows desirability contour plot for multi-objective optimization. Optimal cutting area is the area with the highest composite desirability values. This area is marked in the contour plot with the darkest green color. This optimal cutting area is also visible in Figure 18 as white colored zone.

## 6. Conclusions

This paper presents the investigation of the machinability of the aluminium alloy 5083 by the plasma jet cutting process. The novelty of this study is in introducing a hybrid approach of RSM and desirability analysis as a useful instrument to analyze process responses and to perform simultaneous optimization of multiple cut quality characteristics. Due to its simplicity and reduced time needed for experimenting this approach is widely used in many researches and manufacturing industries to effectively set optimal settings of process parameters. This approach has not been yet presented in the area of analysis and optimization of plasma jet cutting process responses. Experimental data obtained at different cutting speed, arc current, and cutting height were used to develop regression mathematical models to predict cut quality responses: top kerf width, bevel angle, surface roughness *Ra* and *Rz*, and material removal rate. Prediction capabilities of these models were confirmed by MAPE and R^2^ values. ANOVA showed that cutting speed, arc current, and their interactions are significant for all analyzed responses, while for bevel angle response cutting height and interaction cutting height × cutting speed also have significant effect. Desirability analysis was proved as very effective in optimizing simultaneously all analyzed cut quality responses. As main result of multi-objective optimization Pareto front of optimal solutions as well as optimal cutting area were defined. Therefore, in industrial applications operator can set process parameters settings by observing desired values of cut quality characteristics.

In future research more emphasis will be put on application of artificial intelligence methods such as ANN and fuzzy logic in modeling relationships between input process parameters and cut quality responses in plasma jet cutting process of aluminium alloys. Furthermore, significant attention will be put on utilization of evolutionary metaheuristic algorithms in multi-response optimization tasks.

## Figures and Tables

**Table 1:** Summarized literature review.

Material	Process Parameters	Responses	Modelling/Optimization Techn.	Ref.
EN31 steel	GP, AC, torch height	MRR SR	GRA, ANOVA	[1]
St37 carbon steel	CS, SD, arc voltage	Ra, Rz	Taguchi ANOVA	[2]
AISI 316 stainless steel	feed rate, AC, AV, torch height	kerf, chamfer, dross, SR, MRR	RSM, GRA, PCA	[3]
E30 mild steel	AC, CS, CH	BA	Regression analysis, ANOVA, ANOM	[4]
316L stainless steel	thickness, AV, CS, GP	KW, taper	GRA, ANOVA	[5]
304L stainless steel	GP, CS, AC, SD	SR, KW	RSM, GRA, Taguchi	[6]
EN10025 stainless steel	AC, plate thickness, CS	Rz	ANN	[7]
S235JRG2 structural steel	AC, CS	Ra	ANN	[8]
Hardox-400 steel	CS, plasma flow rate, AV	CSU	ANOVA	[9]
Mild steel	CS, AV, plasma gas mass flow rate, shield gas mass flow rate, shield gas mixture	kerf position and shape, CSU	Regression analysis, ANOVA	[10]
St 52-3N steel	CS, AC, GP, CH	Ra, Rtm		[11]
SS420 stainless steel	AC, CS, CH	Ra, MRR	Taguchi, ANOVA, GRA	[12]
St37 mild steel	plate thickness, CS, AC, AV, GP, pierce height, SD	BA	ANN, ANOVA	[13]
EN10025 stainless steel	plate thickness, CS, AC	KW, SR, BA		[14]
AISI 304 stainless steel	CS, material thickness, AC	Ra	RSM, ANOVA	[15]
Titanium	cutting gas, CS	CSU, KW, BA, Rz, HAZ, Temperature		[16]
Monel 400™ alloy	CS, GP, AC, SD	MRR, kerf taper, HAZ width	Regression a., Fuzzy logic, ANOVA, Sensitivity analysis	[17]

Abbreviations: GP—gas pressure, AC—arc current, CS—cutting speed, SD—standoff distance, AV—arc voltage, CH—cutting height, SR—surface roughness, BA—bevel angle, KW—kerf width, CSU—cut surface unevenness, GRA—grey relational analysis, RSM—response surface method, PCA—principal component analysis, ANOM—Analysis of means, ANN—Artificial neural network.

**Table 2:** Specifications of CNC machine FlameCut 2513.

Model	FlameCut 2513
Overall sizes (mm)	3720 × 2500 × 1850
Weight (kg)	1300
Tool travels (X, Y, Z)	3000 mm × 1500 mm × 200 mm
Maximum tool speed	20,000 mm/min
Supply	400 V/50 HZ
Drive	AC Servo
X, Y, Z drive	Ball screw
Accuracy	0.1 mm/m
Plasma type	User defined

**Table 3:** Properties of aluminium EN AW-5083 H111.

**Chemical Composition**								
Al	Ti	Cr	Zn	Si	Mg	Cu	Fe	Mn
Balance	0.15	0.05–0.25	0.25	0.40	4–4.90	0.10	0.40	0.40–1
**Physical Properties**								
Density		Melting point	Thermal expansion		Modulus of elasticity	Thermal conductivity		Electrical resistivity
2.65 g/cm^3^		570 °C	25 × 10^−6^/K		72 GPa	121 W/m·K		0.058 × 10^−6^ Ω·m
**Mechanical Properties**								
Proof stress		Tensile strength	Elongation		Shear strength		Hardness vickers	
145 MPa		300 MPa	23%		175 MPa		75 HV	

**Table 4:** Specifications of Universal Toolmaker’s Microscope.

Model	JX13B
X-coordinate (mm)	200 mm
Y-coordinate (mm)	100 mm
Resolution ration (mm)	X and Y coordinate: 0.0002 mm
Accuracy	(1 + L/100) μm (L—length of workpiece in mm)
Metering force	0.1 N
Locating stability	0.001 mm
Limit error gauged for head diameter	0.0005 mm
Objective magnification	1× 3× 5×
Gross magnifying power	10× 30× 50×
Object working distance	79 mm 69 mm 49 mm
Object visual field	20 mm 6.7 mm 4 mm
Overall sizes (mm)	1300 × 1250 × 800

**Table 5:** Experimental plan and cut quality responses values.

Exp. Trial	Process Parameters							Cut Quality Responses			
	*v* (mm/min)		*I* (A)		*H* (mm)		*Wu* (mm)	*α* (°)	*Ra* (µm)	*Rz*	*MRR* (mm^3^/min)
	Cod.	Real	Cod.	Real	Cod.	Real				(µm)	
1	−1	2000	−1	45	−1	1	2.179	7.782	15.30	66.00	10,614.00
2	−1	2000	−1	45	0	1.5	2.192	6.993	13.00	62.00	10,944.00
3	−1	2000	−1	45	1	2	2.185	6.013	14.60	74.00	11,214.00
4	−1	2000	0	65	−1	1	2.058	3.805	17.70	71.00	11,151.00
5	−1	2000	0	65	0	1.5	2.375	4.745	16.70	59.00	12,756.00
6	−1	2000	0	65	1	2	2.202	2.252	17.40	85.00	12,504.00
7	−1	2000	1	85	−1	1	2.024	3.367	19.30	85.00	13,203.00
8	−1	2000	1	85	0	1.5	2.209	3.633	19.10	76.00	14,397.00
9	−1	2000	1	85	1	2	2.343	1.327	21.60	83.00	14,475.00
10	0	4000	−1	45	−1	1	1.976	9.044	13.20	66.00	17,982.00
11	0	4000	−1	45	0	1.5	2.044	9.722	11.80	53.00	18,360.00
12	0	4000	−1	45	1	2	1.920	8.876	10.10	51.00	17,418.00
13	0	4000	0	65	−1	1	1.944	5.313	7.40	34.00	19,980.00
14	0	4000	0	65	0	1.5	1.849	5.048	9.90	53.00	19,008.00
15	0	4000	0	65	1	2	1.957	5.682	7.70	40.00	19,902.00
16	0	4000	1	85	−1	1	1.993	2.891	13.00	60.00	22,098.00
17	0	4000	1	85	0	1.5	2.020	3.272	11.30	51.00	22,182.00
18	0	4000	1	85	1	2	2.111	2.796	11.80	58.00	23,574.00
19	1	6000	−1	45	−1	1	1.952	11.539	10.60	52.00	24,111.00
20	1	6000	−1	45	0	1.5	2.001	12.662	11.70	52.00	23,886.00
21	1	6000	−1	45	1	2	1.988	10.444	9.60	45.00	25,830.00
22	1	6000	0	65	−1	1	2.242	8.643	8.90	42.00	32,148.00
23	1	6000	0	65	0	1.5	2.166	9.648	6.40	34.00	29,808.00
24	1	6000	0	65	1	2	2.199	9.360	5.60	34.00	30,681.00
25	1	6000	1	85	−1	1	2.296	7.360	5.50	34.00	34,353.00
26	1	6000	1	85	0	1.5	2.377	8.428	5.70	36.00	34,785.00
27	1	6000	1	85	1	2	2.492	7.360	5.30	31.00	37,881.00

**Table 6:** ANOVA for top kerf width.

Source	DF	SS	MS	F-Value	*p*-Value	(%)
*v*	2	4.6374	2.31869	25.58	0.000	40.587
*I*	2	1.8349	0.91745	10.12	0.006	16.059
*H*	2	0.5124	0.25621	2.83	0.118	4.484
*v·I*	4	2.6035	0.65088	7.18	0.009	22.786
*v·H*	4	0.4632	0.11581	1.28	0.355	4.054
*I·H*	4	0.6490	0.16226	1.79	0.224	5.680
Error	8	0.7250	0.09063	-	-	6.345
Total	26	11.4255	-	-	-	-

The standard tabulated value of F-ratio: F_0.05,2,8_ = 4.46, F_0.05,4,8_ = 3.84.

**Table 7:** ANOVA for bevel angle.

Source	DF	SS	MS	F-Value	*p*-Value	(%)
*v*	2	265.579	132.789	78.66	0.000	42.558
*I*	2	243.737	121.869	72.19	0.000	39.058
*H*	2	25.498	12.749	7.55	0.014	4.085
*v·I*	4	37.913	9.478	5.61	0.019	6.075
*v·H*	4	32.129	8.032	4.76	0.029	5.148
*I·H*	4	5.676	1.419	0.84	0.537	0.909
Error	8	13.505	1.688	-	-	2.164
Total	26	624.037	-	-	-	-

The standard tabulated value of F-ratio: F_0.05,2,8_ = 4.46, F_0.05,4,8_ = 3.84.

**Table 8:** ANOVA for surface roughness *Ra*.

Source	DF	SS	MS	F-Value	*p*-Value	(%)
*v*	2	240.243	120.121	98.46	0.000	70.122
*I*	2	13.150	6.575	5.39	0.033	3.838
*H*	2	3.227	1.613	1.32	0.319	0.941
*v·I*	4	69.796	17.449	14.30	0.001	20.372
*v·H*	4	4.606	1.151	0.94	0.486	1.344
*I·H*	4	1.824	0.456	0.37	0.821	0.532
Error	8	9.760	1.220	-	-	2.848
Total	26	342.606	-	-	-	-

The standard tabulated value of F-ratio: F_0.05,2,8_ = 4.46, F_0.05,4,8_ = 3.84.

**Table 9:** ANOVA for surface roughness *Rz*.

Source	DF	SS	MS	F-Value	*p*-Value	(%)
*v*	2	130.384	65.1919	39.70	0.000	68.634
*I*	2	11.893	5.9466	3.62	0.076	6.260
*H*	2	0.892	0.4459	0.27	0.769	0.469
*v·I*	4	25.128	6.2819	3.83	0.050	13.227
*v·H*	4	7.222	1.8055	1.10	0.419	3.801
*I·H*	4	1.314	0.3285	0.20	0.931	0.691
Error	8	13.137	1.6421	-	-	6.915
Total	26	189.969	-	-	-	-

The standard tabulated value of F-ratio: F_0.05,2,8_ = 4.46, F_0.05,4,8_ = 3.84.

**Table 10:** ANOVA for material removal rate.

Source	DF	SS	MS	F-Value	*p*-Value	(%)
*v*	2	271.674	135.837	1416.68	0.000	89.520
*I*	2	27.436	13.718	143.07	0.000	9.040
*H*	2	0.720	0.360	3.76	0.071	0.237
*v·I*	4	1.712	0.428	4.46	0.034	0.564
*v·H*	4	0.896	0.224	2.34	0.143	0.295
*I·H*	4	0.270	0.068	0.70	0.611	0.088
Error	8	0.767	0.096	-	-	0.252
Total	26	303.477	-	-	-	-

The standard tabulated value of F-ratio: F_0.05,2,8_ = 4.46, F_0.05,4,8_ = 3.84.

**Table 11:** Multi-objective optimization results.

Optimized Response	Optimum Process Parameters				Optimum Response Values				Desirability
	*v*	*I*	*H*	*Wu*	*α*	*Ra*	*Rz*	*MRR*	
	(mm/min)	(A)	(mm)	(mm)	(°)	(µm)	(µm)	(mm^3^/min)	
min *Wu*, min *α*, min *Ra*, min *Rz*, max *MRR*	5232.32	80.151	1	2.084	5.751	7.541	39.38	28,985.2	0.717
	4515.35	85	1	2.003	4.413	9.936	48.40	25,687.2	0.682
	6000.00	80.836	1	2.249	7.216	6.014	33.96	34,017.1	0.675
	4395.44	85	1	1.996	4.326	10.31	49.69	25,014.3	0.670
	4368.46	85	1	1.989	4.221	10.42	50.02	24,836.2	0.669

## Data Availability

The data presented in this study are available from the corresponding author, upon reasonable request.

## References

[1] Kumar Das M., Kumar K., Barman K.T., Sahu P. (2014). Optimization of process parameters in plasma arc cutting of EN31 steel based on MRR and multiple roughness characteristics using grey relational analysis. Procedia Mater. Sci..

[2] Tsiolikas A., Kechagias J., Salonitis K., Mastorakis N. (2016). Optimization of cut surface quality during CNC Plasma Arc Cutting process. Int. J. Syst. Appl. Eng. Dev..

[3] Maity K.P., Kumar Bagal D. (2015). Effect of process parameters on cut quality of stainless steel of plasma arc cutting using hybrid approach. Int. J. Adv. Manuf. Technol..

[4] Srinivasa Raju S.V.S.S., Kodanda Ram K., Satyanarayana D.V.S.S., Sai Nishood Goud M. (2016). Optimization of Process Parameters of Plasma Arc Cutting Using Taguchi’s Robust Design Methodology. IOSR J. Mech. Civ. Eng..

[5] Pawar S.S., Inamdar K.H. (2017). Experimental Analysis of Plasma Arc Cutting Process for SS 316L Plates. IOSR J. Mech. Civ. Eng..

[6] Adalarasan R., Santhanakumar M., Rajmohan M. (2015). Application of Grey Taguchi-based response surface methodology (GT-RSM) for optimizing the plasma arc cutting parameters of 304L stainless steel. Int. J. Adv. Manuf. Technol..

[7] Radovanovic M., Madic M. (2011). Modeling the plasma arc cutting process using ANN. Nonconv. Technol. Rev..

[8] Peko I., Nedić B., Đorđević A., Džunić D., Janković M., Veža I. (2016). Modeling of Surface Roughness in Plasma Jet Cutting Process of Thick Structural Steel. Tribol. Ind..

[9] Chamarthi S., Sinivasa Reddy S., Kumar Elipey M., Ramana Reddy D.V. (2013). Investigation Analysis of Plasma arc cutting Parameters on the Unevenness surface of Hardox-400 material. Procedia Eng..

[10] Bini R., Colosimo B.M., Kutlu A.E., Monno M. (2008). Experimental study of the features of the kerf generated by a 200A high tolerance plasma arc cutting system. J. Mater. Process. Technol..

[11] Nedić B., Janković M., Peko I. Surface roughness analysis at plasma cutting. Proceedings of the 15th International Conference on Tribology.

[12] Sandeep R., Sudhakara D., Sreenivasulu B. (2015). Multi objective optimization of process parameters in plasma arc cutting of SS 420 using Grey-Taguchi analysis. Int. J. Adv. Eng. Res. Sci..

[13] Kechagias J., Pappas M., Karagiannis S., Petropoulos G., Iakovakis V., Maropoulos S. An ANN Approach on the Optimization of the Cutting Parameters during CNC Plasma Arc-Cutting. Proceedings of the 10th Biennial Conference on Engineering Systems Design and Analysis.

[14] Lazarević A. (2014). Experimental research of the plasma arc cutting process. J. Appl. Eng. Sci..

[15] Ilii S.M., Coteata M., Munteanu A. (2010). Experimental results concerning the variation of surface roughness parameter (Ra) at plasma arc cutting of stainless steel workpiece. Int. J. Mod. Manuf. Technol..

[16] Gariboldi E., Previtali B. (2005). High tolerance plasma arc cutting of commercially pure titanium. J. Mater. Process. Technol..

[17] Devaraj R., Abouel Nasr E., Esakki B., Kasi A., Mohamed H. (2020). Prediction and Analysis of Multi-Response Characteristics on Plasma Arc Cutting of Monel 400TM Alloy Using Mamdani-Fuzzy Logic System and Sensitivity Analysis. Materials.

[18] Peko I., Nedić B., Đorđević A., Veža I. (2018). Modelling of Kerf Width in Plasma Jet Metal Cutting Process using ANN Approach. Tech. Gaz..

[19] Peko I., Nedić B., Dunđer M., Samardžić I. (2020). Modelling of Dross Height in Plasma Jet Cutting Process of Aluminium Alloy 5083 Using Fuzzy Logic Technique. Tech. Gaz..

[20] Kadirgama K., Noor M.M., Harun W.S.W., Aboue-El-Hossein K.A. (2010). Optimisation of heat affected zone by partial swarm optimisation in air plasma cutting operation. J. Sci. Ind. Res..

[21] Peko I., Nedić B., Đorđević A., Đurić S., Džunić D., Veža I., Janković M. Prediction of surface roughness in plasma jet cutting process using neural network model. Proceedings of the 15th International Conference on Tribology.

[22] Peko I., Nedić B., Dunđer M., Samardžić I. (2020). Taguchi optimization of bevel angle in plasma jet cutting process of aluminium alloy 5083. J. Prod. Eng..

[23] Hamid A., Novareza O., Dwi Widodo T. (2019). Optimization of process parameters and quality results using plasma arc cutting in aluminium alloy. J. Eng. Manag. Ind. Syst..

[24] Patel S.B., Vyas T.K. Parametric Investigation of Plasma Arc Cutting on Aluminium Alloy 6082. Proceedings of the International Conference on Ideas, Impact and Innovation in Mechanical Engineering.

[25] Madić M., Radovanović M., Manić M., Trajanović M. (2014). Optimization of CO2 Laser Cutting Process using Taguchi and Dual Response Surface Methodology. Tribol. Ind..

[26] Kuntoglu M., Acar O., Kumar Gupta M., Saglam H., Sarikaya M., Giasin K., Yurievich Pimenov D. (2021). Paramateric Optimization for Cutting Forces and Material Removal Rate in the Turning of AISI 5140. Machines.

[27] Rahali El Azzouzi S., Kobi A., Barreau M. (2013). Multi-Response Optimization For Industrial Processes. Int. J. Eng..

[28] Hussein W., Abdullah Al-Shammari M. (2018). Fatigue and Fracture Behaviours of FSW and FSP Joints of AA5083-H111 Aluminium Alloy. IOP Conf. Ser. Mater. Sci. Eng..

[29] Peko I., Ljumović P., Nedić B., Dunđer M. (2019). Analysis of the heat affected zone in plasma jet cutting process of the aluminium alloy EN AW 5083. Zaštita Mater..

[30] Cueca F., Solano E., Patarroyo A., Morales A., Rojas F., Muñoz R. (2012). Study of the weld ability of Aluminum Alloy 5083 H116 with Pulsed Arc GMAW (GMAW-P). Ship Sci. Technol..

[31] Hernández-Castillo I., Sánchez-López O., Lancho-Romero G.A., Castañeda-Roldán C.H. (2018). An experimental study of surface roughness in electrical discharge machining of AISI 304 stainless steel. Ing. Investig..

[32] Lee W.K., Abdullah M.D., Ong P., Abdullah H., Teo W.K. (2021). Prediction of flank wear and surface roughness by recurrent neural network in turning process. J. Adv. Manuf. Technol..

[33] Madić M., Radovanović M., Trajanović M., Manić M. (2014). Analysis of correlations of multiple-performance characteristics for optimization of CO2 laser nitrogen cutting of AISI 304 stainless steel. J. Eng. Sci. Technol. Rev..

[34] Madić M., Radovanović M., Nedić B., Marušić V. (2015). Multi-objective optimization of cut quality characteristics in CO_2_ laser cutting of stainless steel. Tech. Gaz..

[35] Madić M., Radovanović M. (2012). An artificial intelligence approach for the prediction of surface roughness in CO_2_ laser cutting. J. Eng. Sci. Technol..

[36] Derringer G., Suich R. (1980). Simultaneous optimization of several response variables. J. Qual. Technol..

[37] Sidhu H.S., Banwait S.S. (2014). Analysis and Multi-objective Optimisation of Surface Modification Phenomenon by EDM Process with Copper-Tungsten Semi-sintered P/M Composite Electrodes. Am. J. Mech. Eng..

[38] Minitab 18 Support. https://support.minitab.com/en-us/minitab/18/help-and-how-to/modeling-statistics/using-fitted-models/supporting-topics/response-optimization/determining-the-weight/.

[39] Minitab 18 Support. https://support.minitab.com/en-us/minitab/18/help-and-how-to/modeling-statistics/using-fitted-models/supporting-topics/response-optimization/what-is-importance-in-response-optimization/.

[40] Kusumoto K., Wang J., Nezu K. (1999). A Study on the Cut Surface Quality of Mild Steel Plate by Oxygen Plasma Arc Cutting. Q. J. Jpn. Weld. Soc..

[41] Wang J., Zhu Z., He C., Yang F. (2011). Effect of dual swirling plasma arc cutting parameters on kerf characteristics. Int. J. Mater. Form..

